# Health-related quality of life in patients after endoscopic or microscopic cholesteatoma surgery

**DOI:** 10.1007/s00405-024-09097-8

**Published:** 2024-11-27

**Authors:** Yannik Raemy, David Bächinger, Nicole Peter, Christof Roosli

**Affiliations:** Department of Otorhinolaryngology, Head and Neck Surgery, University Hospital Zürich, University Zürich, Frauenklinikstrasse 24, 8091 Zürich, Switzerland

**Keywords:** Cholesteatom, Endoscopic ear surgery, Microscope, Quality of life, ChOLE staging

## Abstract

**Purpose:**

Different surgical techniques exist for treating cholesteatoma, such as microscopical or transcanal endoscopic ear surgery (TEES). This study aimed to compare these two techniques, focusing on quality of life.

**Methods:**

This retrospective single-center study included 188 patients with cholesteatoma. The primary outcome was the assessment of health-related quality of life (HRQoL) using the Zurich Chronic Middle Ear Inventory (ZCMEI-21) preoperatively, 3 and 12 months postoperatively with regard to surgical technique and intraoperative staging of the cholesteatoma (ChOLE classification). Secondary outcomes included hearing pure tone average of 0.5, 1, 2 and 4 kHz (PTA_4_), complications assessed 3 months postoperatively as well as recidivism within the follow-up time of 1 year postoperatively.

**Results:**

A total of 28 patients underwent TEES and 160 microscopic ear surgery. The ZCMEI-21 total scores preoperatively were not significantly different between the two groups. An improvement in QoL one year postoperatively was observed in both groups to a comparable extent. The ZCMEI-21 decreased significantly (*p* < 0.01) in both groups. In the TEES group, the cholesteatoma tended to be smaller (lower ChOLE score), PTA_4_ was better and complication rate comparable. The number of recidivisms was lower for the TEES group (1 [3.6%] vs. 31 [19.4%]).

**Conclusion:**

TEES is a valuable alternative to the traditional microscopic technique, at least for small cholesteatoma, which leads to a comparable improvement in HRQoL as the microscopic technique. The better postoperative hearing and lower rate of recidivism in the TEES group may be related to the smaller extent of the cholesteatoma.

## Introduction

Patients with chronic otitis media with cholesteatoma (COMC) typically suffer from hearing impairment, fetid ear discharge and sometimes pain, tinnitus, or vertigo. Various studies show that all these symptoms have an impact on health-related quality of life (HRQoL), at least to some extent.

Surgical therapy is indicated for patients with COMC. Since its introduction in 1951, the microscope became the gold standard for otosurgery because of its ability to magnify and illuminate the surgical field [1]. It also allows simultaneous work with both hands. A limitation of the microscope is the pure ergonomics due to the fixed optical axis and the straight-line view. Since the 1990s, endoscopic ear surgery has become widely used because it avoids a retroauricular incision and has less cosmetic impact on the patient. It also provides access to hidden areas using angulated lenses with a wide and clear view of the surgical field. Image resolution is excellent [2]. Often, less healthy tissue needs to be disrupted for access compared to a microscopic access. However, in TEES, typically only one hand is available for manipulation, as the other is occupied with holding the camera. Further, stereoscopic view is lacking, and a relatively long surgical learning curve exists. A third option is the use of an exoscope that allows bimanual work but improves ergonomic limitations of the microscope and endoscope that may have an impact on the surgeon [3].

The outcome after surgery can be assessed in several ways taking different parameters into consideration. Objective aspects such as hearing before and after surgery can be measured relatively easily by comparing hearing threshold before and after surgery. Complications due to an intervention can be assessed from the patients’ charts prospectively, or even retrospectively. Other aspects including subjective factors that affect quality of life need to be assessed specifically [4]. Even after surgical treatment of COMC, HRQoL may be reduced. Since HRQoL has become increasingly important in recent years, various tools have been developed to determine it. Specifically designed and validated questionnaires are typically used to measure patient-reported outcomes. One of these instruments to objectively assess HRQoL in patients with COMC, is the Zurich Chronic Middle Ear Inventory 21 (ZCMEI-21), that is validated in several languages [5]. It concerns four different aspects, including ear signs and symptoms, hearing, psychosocial impact, and medical resources [6].

Comparison of microscopic and endoscopic treatment of COMC show comparable results regarding hearing, complications such as taste disturbance, dizziness, pain and healing time [7–9]. However, little is known about the effect of surgical technique on postoperative HRQoL. The effect of endoscopic surgery for COMC on HRQoL is not evaluated in detail by comparing preoperative and postoperative HRQoL.

Therefore, the aim of study is to compare HRQoL before and after surgery for COMC with regard to surgical technique. Additionally, hearing outcome and complication rate are evaluated. Further the endoscopic and microscopic techniques are compared.

## Method

This study was approved by the Ethic Committee (Nr. 2021_00902) and informed consent was obtained from all subjects and parents of the tested infants. The study was performed according to the Declaration of Helsinki [10].

### Subjects

This study included 370 ears of 341 patients who underwent endoscopic or microscopic cholesteatoma surgery between April 2016 and June 2021 at a tertiary referral center. The data were prospectively collected and retrospectively analyzed. Inclusion criteria were age > 18 years, no previous ear surgery on the ear analyzed, no mental disease or cognitive impairment (i.e. dementia), and no previous disease or treatment that may have affected hearing (i.e. ototoxic medication, barotrauma). Of the 370 ears, the following were excluded: previous ear surgery on the affected side (*n* = 81), missing or denial of informed consent (*n* = 75), and inability to fill in the questionnaire (*n* = 26). Eventually 188 ears (182 patients) were analyzed, 28 (28 patients) with endoscopic surgery and 160 (154 patients) with microscopic surgery.

### Hearing thresholds

Hearing thresholds were determined on both sides with a clinical audiometer (Equinox 2.0; Interacoustics, Middelfart, Denmark) and headphones (HDA 300; Sennheiser, Wedemark, Germany) in a sound-isolated booth. For air conduction (AC) the 0.25, 0.5, 0.75, 1, 2, 3, 4, 6 and 8 kHz were considered, while for bone conduction (BC) the frequencies of 0.5, 1, 2 and 4 kHz were measured. The threshold measurement procedure was based on a conventional bracketing method to obtain responses with a step size of 5 dB, and participants responded by pressing a response button. The hearing threshold was measured preoperatively (within 3 months before surgery), 3 months and 12 months postoperatively.

Pure tone average (PTA_4_) was calculated with the average of the values at 0.5 kHz, 1 kHz, 2 kHz and 4 kHz. The Air-Bone Gap (ABG) refers to the difference between AC PTA_4_ and BC PTA_4_.

### Health-related quality of life

The ZCMEI-21 [5] was used preoperatively, 3 months and 12 months postoperatively to assess HRQoL. It is a validated tool to assess disease-specific quality of life in patients with chronic middle ear disease has been translated in several languages [6]. Its use includes the clinical settings as well as research and clinical practice [5]. The ZCMEI-21 consists 21 items with a with response option on a 5-point Likert scale ranging from 0 to 4. The maximum score is 84 with high scores correlating with a poorer quality of life. The questionnaire can be divided into four subscales concerning ear signs and symptoms, hearing function, psychosocial impact and the use of medical resources. The minimal clinical important difference (MCID) is estimated to 5 [11].

### Cholesteatoma staging

The ChOLE classification was used to classify and stage the cholesteatomas to allow comparison of different size and extent of the cholesteatoma, differences in the state of the middle ear ossicles, perioperative complications and pneumatisation of the mastoid [12]. These four different aspects of cholesteatoma are considered in the ChOLE classification [13]: (i) extension (ranging from subdivisions Ch1 describing a very limited extension within the middle ear to Ch4 describing a petrous apex cholesteatoma); (ii) the status of the ossicular chain (with O0 indicating an intact ossicular chain to O4 indicating an ossicular defect with only remaining a fixed stapes or stapes footplate. Of note, the O subdivision describes the status of the ossicular chain after removal of the cholesteatoma, but before ossicular chain reconstruction); (iii) complications from L0 (no complications), L2 (extracranial complications, and L4 (intracranial complications); and (iv) determination of Eustachian tube function, defined by the degree of mastoid pneumatization and ventilation ranging from E0 (good pneumatisation and ventilation) to E2 (poor pneumatization and ventilation). Based on the subdivisions of these four aspects, a staging into three different stages (I–III) is undertakten using a numeric rule. The ChOLE classification was determined based on intraoperative findings (subdivisions Ch, O and L) and preoperative CT imaging (subdivision E) using a freely available online software tool [13].

### Recidivism and postoperative complications

Recidivism (recurrence of persistence) of the cholesteatoma on the operated side within the follow-up time of 1 year was evaluated. Both recurrent and persistent cholesteatoma are summarized in the term recidivisms because it is often difficult to distinguish between the two.

The following postoperative complications were analysed:

- Bleeding defined as a situation that required medical intervention within one month after surgery.
- Infection in the surgical field within 3 months after surgery that required medical intervention.
- New occurrence of increase of dizziness, tinnitus, complete sensorineural hearing loss, facial nerve injury, or taste disturbance.

### Statistics

Statistical analysis and figure preparation were performed with GraphPad Prism 8.0 (GraphPad Software, San Diego, USA). All possible subjects at the University Hospital of Zurich were included. Therefore, no sample size was calculated. Normal distribution was assessed visually using quantile-quantile plots. If normality could not be determined this way, the D’Agostino-Pearson normality test was used to confirm the Gaussian distribution [14].

The difference in 2 continuous variables with a normal distribution was analyzed with a paired sample t-test. Since the record contains missing values, a mixed effect model was used for the analysis. This mixed effect model uses a compound symmetry covariance matrix and is fit using Restricted Maximum Likelihood. With data records missing completely at random, the results can be interpreted like repeated measures analysis of variance (ANOVA). Additionally, the Geisser-Greenhouse correction was implemented [15].

All tests were 2 sided and a P-value of less than 0.05 was considered significant. The data are shown as mean ± standard deviation unless otherwise stated.

## Results

The patient characteristic of the 188 ears (182 patients) included (160 in the microscopic group, 28 in the endoscopic group) are shown in Table 1 with a much larger group of patients undergoing microscopic surgery. The mean age was not significantly different with 39.9 years (SD 20.3 years) for the microscopic and 37.9 years (SD 21.3 years) for the endoscopic group (*p* = 0.8). The endoscopic group included 67% female (19/28) and 57% right ears (16/28), while the microscopic group consisted of 40% female (64/160) and 49% right ears (78/160).

**Table 1:** Patient characteristics of the two groups including ChOLE-stage

	Microscopic (*n* = 160)	Endoscopic (*n* = 28)	*p*
Age (mean)	39.9 ± 20.4	37.9 ± 21.3	> 0.05
Female	96 (42.5%)	19 (51.4%)	
Right ear	78 (48.8%)	16 (57.1%)	
Left ear	82 (51.2%)	12 (42.9%)	
ChOLE-stage:			
1	36 (22.5%)	25 (89.3%)	
2	116 (72.5%)	3 (10.7%)	
3	2 (1.25%)	0 (0%)	
4	0 (0%)	0 (0%)	

### Hearing

**Fig. 1:**
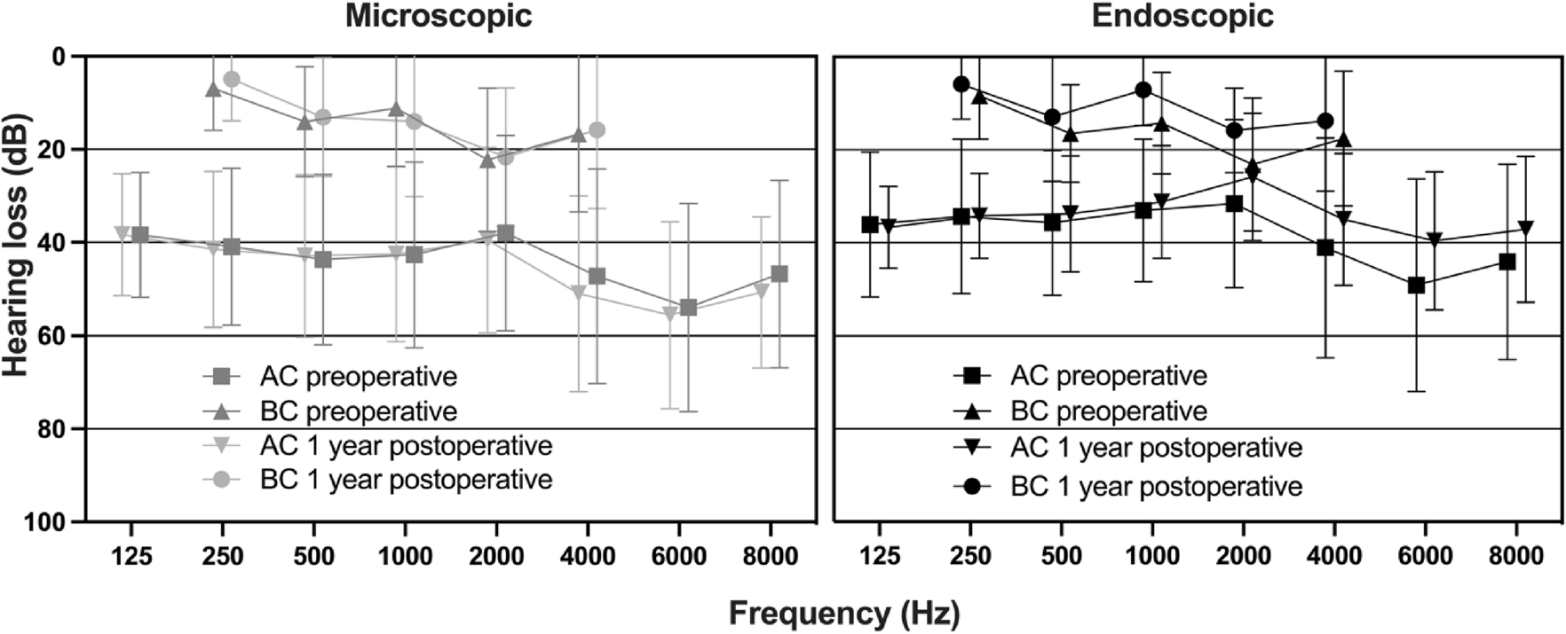
Preoperative and postoperative hearing thresholds are shown for the microscopic group on the left and for the endoscopic group on the right. Preoperative thresholds are shown with solid lines, with squares for BC and circles for AC. Postoperative thresholds are shown with dashed lines, with downward pointing triangles for BC and upward pointing triangles for AC. No statistically significant differences for preoperative and postoperative thresholds were found, nor for the microscopic versus endoscopic group

**Fig. 2:**
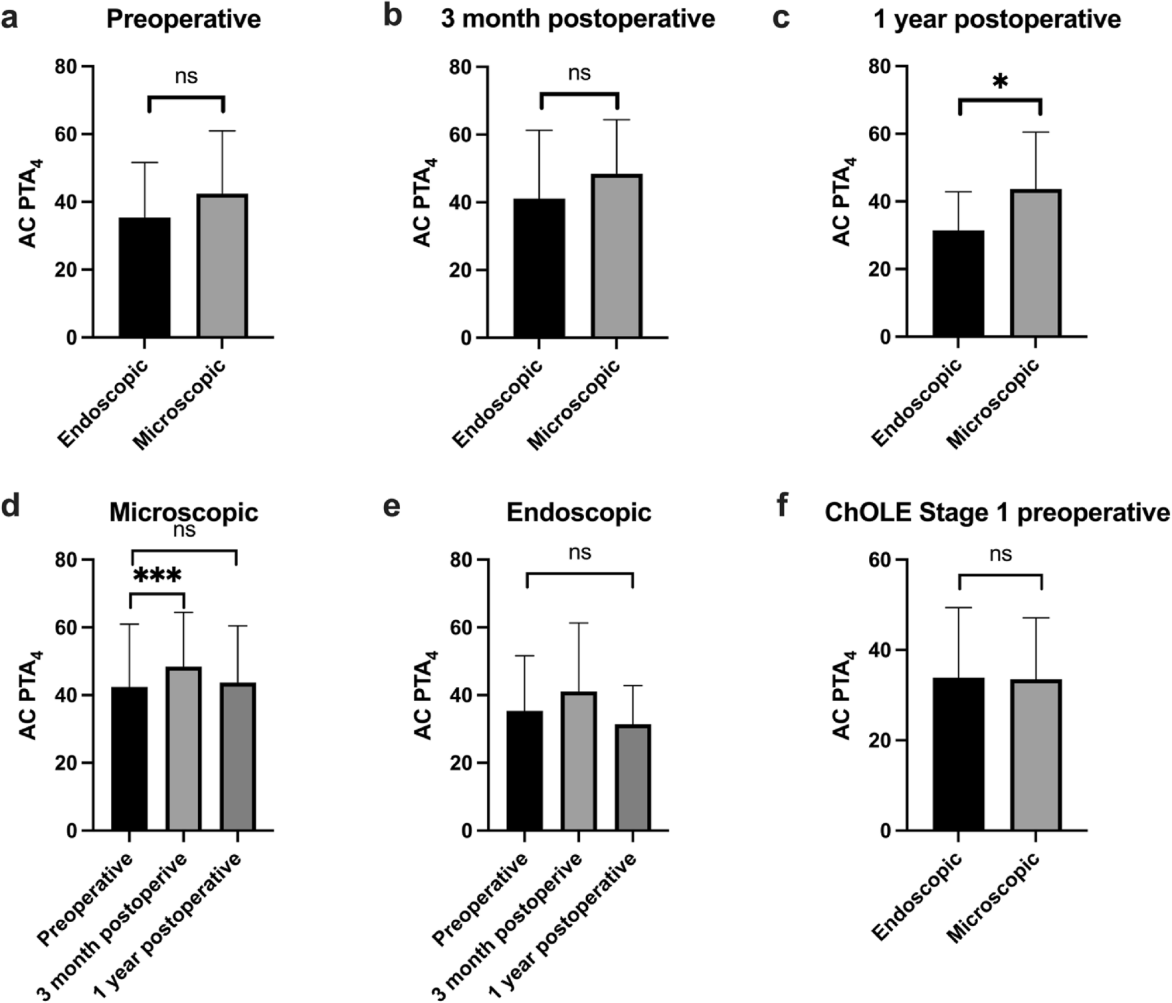
AC PTA_4_ for the endoscopic (black) and microscopic group (grey) is shown in the top row preoperatively (**a**), 3 months postoperatively (**b**), and 1 year postoperatively (**c**). A significant difference between the groups is found 1 year postoperatively with a lower AC PTA_4_ for the endoscopic group The bottom row compares AC PTA_4_ over time for the microscopic group (**d**) and the endoscopic group (**e**). A significant difference of AC PTA_4_ was seen in the microscopic group for preoperative versus 3 months postoperative AC PTA_4_ for stage I cholesteatoma is shown (**f**) with no statistically significant difference between the endoscopic and microscopic groups

The preoperative and one-year postoperative AB and BC hearing thresholds are shown in Fig. 1 for the microscopic approach as well as for the endoscopic approach. While the one-year postoperative BC threshold tended to show slightly better thresholds, especially in the endoscopic group, the differences were not significantly different. The one-year postoperative AC thresholds were slightly worse in the microscopic group in the frequencies > 1 kHz, while the one-year postoperative AC thresholds were slightly better for the endoscopic group > 1 kHz. The comparison of the AC PTA_4_ between the two groups indicated a larger PTA_4_ (worse level of AC PTA_4_) for the microscopic group preoperatively, 3 month and 1 year postoperatively. These differences were statistically significant only 1 year postoperative (Fig. 2, top row). A significant increase of the AC PTA_4_ between preoperative and 3 months postoperative was seen for the microscopic group, while no statistically significant differences over time were seen in the endoscopic group (Fig. 2, bottom row).

Patient with a ChOLE stage I showed no difference of the preoperative AC PTA4 between the endoscopic and microscopic group.

### Health-related quality of life

**Fig. 3:**
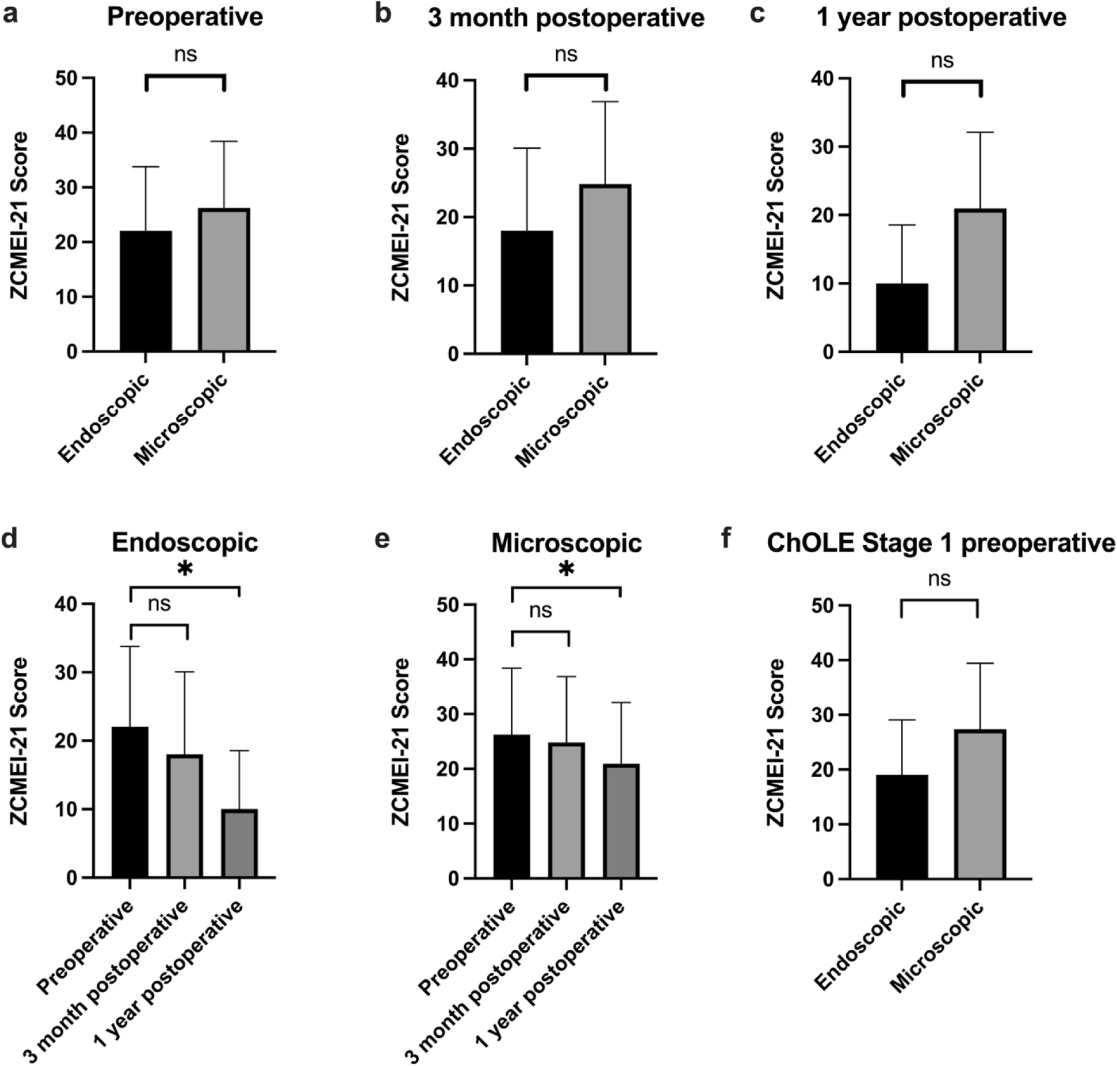
The ZCMEI-21 total score for the endoscopic (black) and microscopic group (grey) is shown in the top row preoperatively (**a**), 3 months postoperatively (**b**), and 1 year postoperatively (**c**). The ZCMEI-21 total scores tended to be lower (better HRQoL) in the endoscopic group at all three time points. However, these differences are not statistically significant The bottom row indicates a decreasing ZCMEI-21 total scores over time for both groups (**d**, **e**) with significant difference between preoperative and one-year postoperative evaluation for both groups ZCMEI-21 total scores for stage I cholesteatoma are shown (**f**) with no significant differences between the two groups

The ZCMEI-21 was completed in the endoscopic group in 15/28 patients (53.5%) preoperatively, in 12/28 patients (43%) 3 month postoperatively and in 5/28 patients (18%) 1 year postoperatively. For the microscopic group, the respective values were 48/160 (30%) preoperatively, 33/160 (20%) 3 months postoperatively, and 17/160 (10.5%) 1 year postoperatively. The total ZCMEI-21 score tended to be lower (better HRQoL) in the endoscopic group at all three time points (preoperatively, 3 months and 1 year postoperatively), but the differences were not statistically significant (Fig. 3). However, over time the total ZCMEI-21 score decreased and showed a significant difference 1 year postoperatively compared to preoperatively for both groups (Fig. 3).

### ChOLE staging

**Fig. 4:**
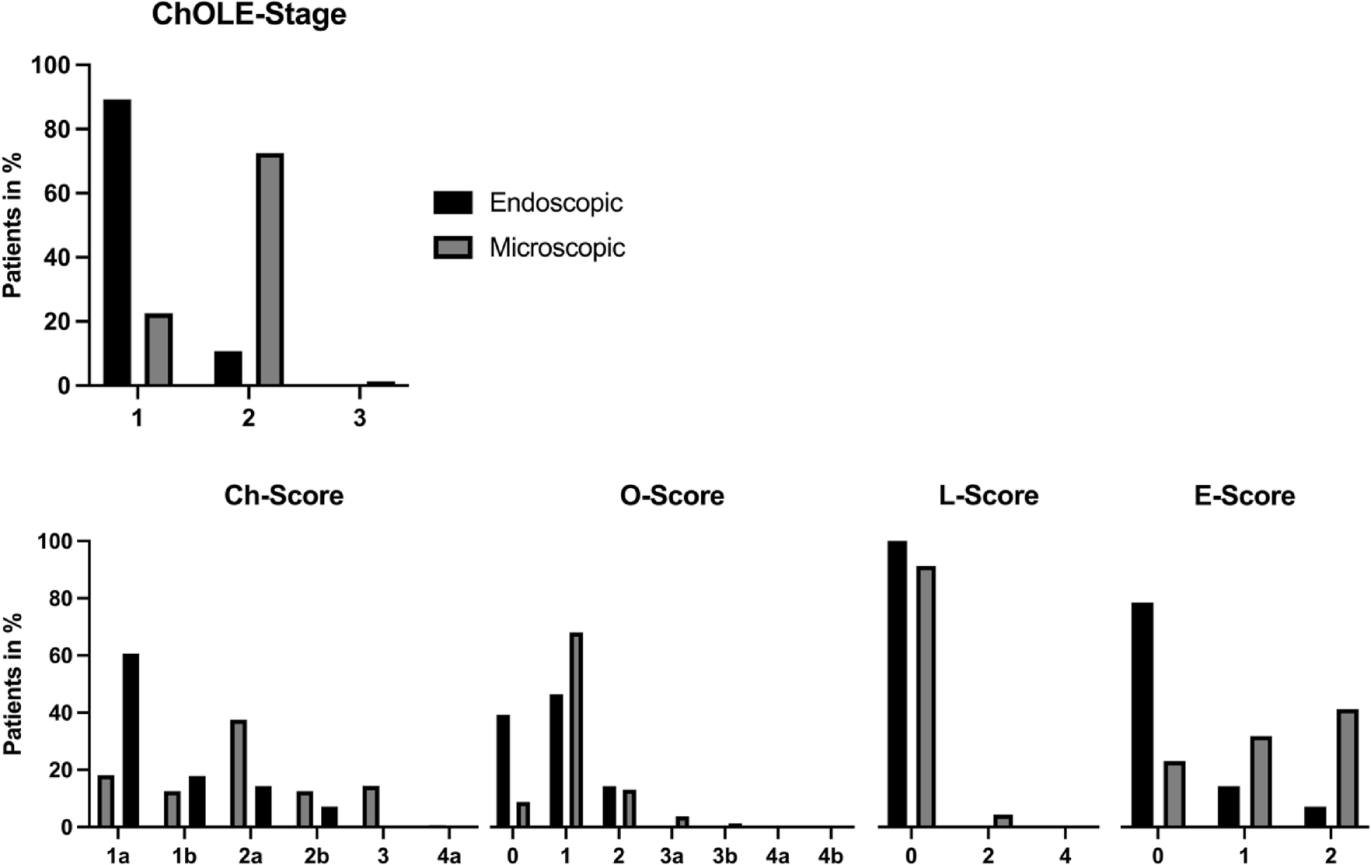
This figure shows the ChOLE stage in the top row for the endoscopic approach in red and the microscopic approach in blue. The score is lower for the endoscopic group The bottom row shows the subscores (Ch, O, L, and E) for the endoscopic (black) and microscopic (grey) group. Ch, O and E score is lower for the endoscopic group, while the L score is comparable. This indicates a smaller extent, less ossicular destruction, and better pneumatization of the endoscopically treated patients

The ChOLE staging is displayed in Fig. 4. The overall comparison showed a much higher number of ChOLE stage I in the endoscopic group (89.3% (*n* = 25) vs. 22.5% (*n* = 36)) while the number of ChOLE stage II was higher in the microscopic group (72.5% (*n* = 116) vs. 10.7% (*n* = 3)). The differences come mainly from the smaller extent of the cholesteatoma (lower Ch score). A Ch score of 1 was found in 22/28 patients (78.5%) in the endoscopic group, and in 49/160 patients (30.6%) in microscopic group. A Ch score of 2 was found in 6/28 patients (21.5%) in the endoscopic group and in 80/160 patients (50%) in the microscopic group. An intact ossicular chain (O score of 0) was found in 11/28 patients (39%) the endoscopic group, and in 14/160 patients (8.7%) in the microscopic group. Further, pneumatization was better in the endoscopic group (lower E score) with 22/28 patients (78.5%) with an E0 score and 4/22 patients (14%) with an E1 score, while in the microscopic group the E0 score was found in 37/160 patients (23%), an E1 score in 51/160 patients (32%), and an E2 score in 66/160 patients (41%).

The L score, indicating complications due to the cholesteatoma is comparable between the groups.

#### Recidivism and postoperative complications

Only one patient (3.6%) in the endoscopic group showed recidivism, while 31 (19.4%) in the microscopic group showed recidivism.

The number of complications is shown in Table 2. The number of complications was 10 in the endoscopic group and 58 in the microscopic group. There are no significant differences in the number of complications between the groups. Tinnitus was the most common complication with 6 patients (21%) in the endoscopic group and 27 patients (16.9%) in the microscopic group. Further complications in the endoscopic group were taste disorder (*n* = 2), infection (*n* = 1) and dizziness (*n* = 1). In the microscopic group, 15 patients developed an infection, 7 patients complained of taste disorders, 7 of dizziness, 1 was revised in the operating theater because of bleeding, and 1 patient developed a transient facial palsy. In general, none of these complications resulted in a long-term impairment.

**Table 2:** Total complications for the two groups as well as number of the evaluated complications are displayed

	Endoscopic (*n* = 28)	Microscopic (*n* = 160)
Total complications	10 (35.7%)	58 (36.3%)
Bleeding	0 (0%)	1 (0.6%)
Infection	1 (3.6%)	15 (9.4%)
Dizziness	1 (3.6%)	7 (4.4%)
Tinnitus	6 (21.4%)	27 (16.9%)
Deafness	0 (0%)	0 (0%)
Transient facial palsy	0 (0%)	1 (0.6%)
Taste disorder	2 (7.1%)	7 (4.4%)

### Comparison of ChOLE stage vs. ZCMEI-21 total score

Subjects of the endoscopic group with a higher ChOLE-score tend to have a higher ZCMEI-21 total score. In the microscopic group the trend seems to be reversed. However, there is no significant difference between the two groups. The number of people with a high ChOLE score is small in both groups and only subjects of the microscopic group had cholesteatoma stage 3 (Table 1).

**Fig. 5:**
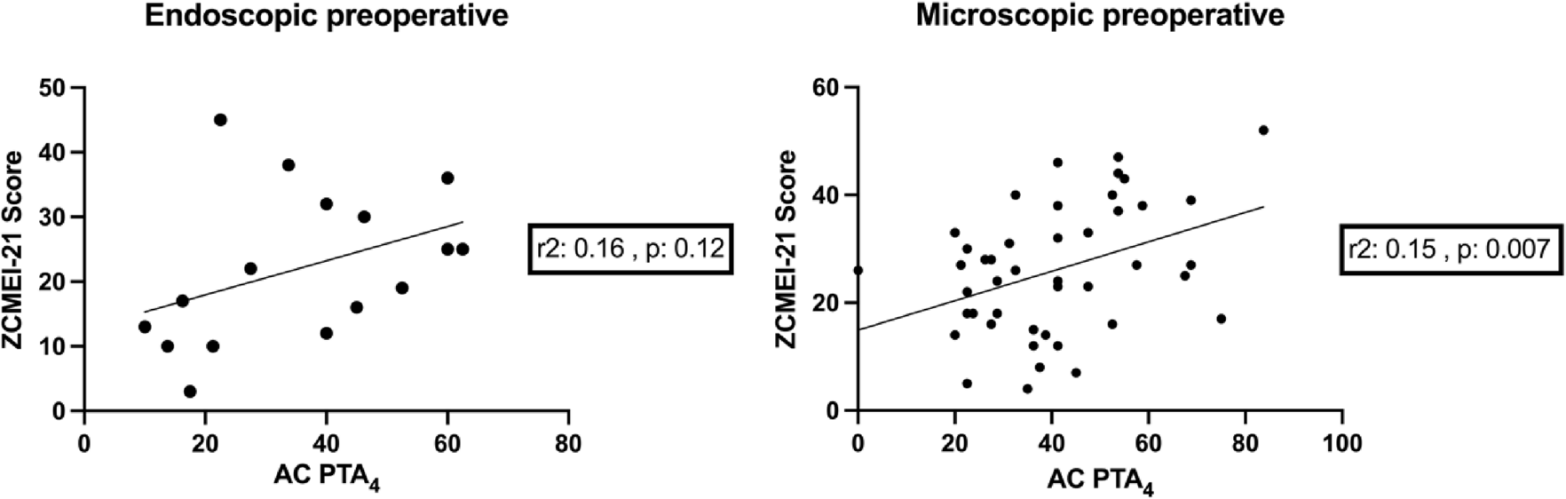
Correlation of hearing (AC PTA_4_) and HRQoL (ZCMEI-21 score) preoperatively. On the left side the endoscopic group is shown. A non significant, positive correlation is shown, resulting in a higher ZCMEI-21 score for poorer hearing. The same, albeit significant, is seen on the right side for the microscopic group

In both, the microscopic and the endoscopic group, HRQoL decreases with poorer hearing. While in the endoscopic group only a trend is visible, the microscopic group shows a significant correlation (Fig. 5).

## Discussion

Microscopic surgical removal of cholesteatoma was the state of the art intervention in treatment of COMC for many years [16]. The use of endoscopic techniques [9], exoscopes [17] or endoscopically assisted microscopic surgery [18] is increasingly described in the last two decades. All surgical techniques aim at complete removal of the disease to prevent recidivism. The term recidivism subsumes residual and recurrent cholesteatoma. A second goal of surgery is to maintain or improve hearing. Finally, the intervention intents to result in a good quality of life. While many studies report on recidivism and/or hearing outcome, there is no clear trend of superiority of one or the other surgical approach [19–22].

The direct comparison between different techniques remains difficult to achieve because patients need to be randomized to treatment, and an experienced surgeon for all techniques needs to be available. Further, the extent of the cholesteatoma affects outcome with a higher rate of recidivism and hearing with larger extent of the disease [23, 24]. The reported follow-up time may also affect the outcome with lower recidivism in investigations with a shorter follow-up time [25]. In the present study, the number of recidivisms is within the range of others for cholesteatoma surgery [20, 26]. The larger number of recidivisms in the microscopic group may correspond to the larger extent of cholesteatoma as shown using the ChOLE classification. This classification allows to correlate the extent of the disease to postoperative results [27]. It also clearly demonstrates that the two groups are not identical and no direct comparison between endoscopic and microscopic surgical technique can be made.

Postoperative hearing is reported one year after surgery representing a longer follow-up than many studies with comparable results to others [28, 29]. Two aspects need to be distinguished: BC and AC thresholds. When it comes to BC, mean hearing thresholds did not become worse 1 year postoperatively compared to preoperatively as shown in Fig. 2. While no patient developed complete sensorineural hearing loss, others may experience a reduction in the Carhart notch, the BC threshold around 2 kHz, as a side effect of reduction in conductive hearing loss. However, mean conductive hearing loss remained the same in both patient groups. The most likely reason is that primary reconstruction of the ossicular chain was rarely performed, and a second look was foreseen. In the meantime, this approach was changed at our institution to a primary reconstruction of the ossicular chain due to the wide availability of Non-EPI Diffusion MRI for follow-up. The slightly better hearing and lower conductive component in the endoscopic group may be related to the smaller extent of the cholesteatoma and to less destruction of the ossicular chain.

An argument to favor an endoscopic approach in surgical treatment of COMC is besides a better visualization of the middle ear structures a less invasive surgical approach [7, 30]. However, quality of life is not systematically assessed. The results of the ZCMEI-21 showed better HRQoL for the patients who underwent endoscopic surgery. This may also correspond to a smaller extension of the disease resulting in less symptoms, to a smaller degree of ossicular destruction. A slightly better hearing (Fig. 2) was found in the endoscopic group. Although the differences were not statistically significant, they could have an effect on everyday life.

A limitation of the evaluation of HRQoL is first of all the lack of randomization, but also the rather low response rate, especially one year after surgery. However, the approach of resecting small COMC endoscopically and larger COMC microscopically resulted in comparable results with small differences on hearing (Figs. 2 and 3), HRQoL (Fig. 4). This indicates that an individual, patient tailored approach is reasonable. To increase the number of patients filling out a HRQoL questionnaire, more effort is required, which may be supported by the automatized sending of the questionnaires, as foreseen for the future in our department.

## Conclusions

An individual, patient tailored approach is reasonable approach of resecting small COMC endoscopically and larger COMC microscopically results in comparable results with slightly better outcome for the endoscopic approach. However, since no randomization of treatment was done, the COMC treated endoscopically were smaller (lower Ch score in the ChOLE-classification). Therefore, no direct comparison between the two surgical techniques can be made.

## Abbreviations

AC: Air conduction
BC: Bone conduction
COMC: Chronic otitis media with cholesteatoma
dB: Decibels
HRQoL: Health-related quality of life
kHz: Kilohertz
MCID: Minimal clinical important difference
PTA: Pure tone average
QoL: Quality of life
TEES: Transcanal endoscopic ear surgery
ZCMEI-21: Zurich chronic middle ear inventory
